# Digital Twin Meets the Bench: Natural Compounds Reshape the Ovarian Cancer Microenvironment

**DOI:** 10.3390/biomedicines13123119

**Published:** 2025-12-18

**Authors:** Anna Kleczka, Radosław Dzik, Agata Kabała-Dzik

**Affiliations:** 1 Department of Pathology, Faculty of Pharmaceutical Sciences in Sosnowiec, Medical University of Silesia in Katowice, Ostrogórska 30, 41-200 Sosnowiec, Poland; adzik@sum.edu.pl; 2 Department of Clinical Engineering, Academy of Silesia, Rolna 43, 40-555 Katowice, Poland

**Keywords:** CAPE, caffeic acid phenethyl ester, ovarian cancer, teratocarcinoma, tumour microenvironment, decorin, CA-72.4, ELISA, digital twin, virtual modelling

## Abstract

**Background**: Malignant ovarian tumours are most often detected at an advanced stage, when peritoneal dissemination across abdominal organs is already present. Metastasis in ovarian cancer arises from complex interactions between cancer cells and diverse components of the tumour microenvironment (TME), including extracellular matrix elements, fibroblasts, adipocytes, mesenchymal cells and leukocytes. This dynamic niche drives tumour progression, invasiveness and immunosuppression through cytokine- and chemokine-mediated signalling. A deeper understanding of these interactions may enable targeted modulation of the TME and help limit metastatic spread. **Methods**: In this study, using immunoenzymatic assays and a computational digital twin—a mechanistic, ODE-based in silico model that replicates key cellular and microenvironmental processes—we investigated whether and how caffeic acid phenethyl ester (CAPE) influences TME activation, cytokine and growth factor levels, and extracellular matrix remodelling. **Results**: Our findings show that CAPE modulates both pro- and antitumourigenic signalling pathways across immune, stromal and hypoxia-related axes, suggesting its potential to reshape the ovarian cancer microenvironment and improve therapeutic outcomes in this challenging malignancy. **Conclusions**: Taken together, these results indicate that CAPE may serve as a multifaceted modulator capable of simultaneously targeting tumour cells and their microenvironment, offering a promising avenue for enhancing therapeutic strategies in ovarian cancer.

## 1. Introduction

Traditional and academic medicine have used natural compounds for centuries because many plant- and animal-derived substances now serve as the foundation for contemporary pharmacological treatments [1]. The biological compounds in propolis include flavonoids and phenolic acids and esters which include caffeic acid (CA) and its phenethyl ester (CAPE), coumaric acid, apigenin, chrysin, galangin, kaempferol, quercetin, genistein, naringin and many others [2]. The two main compounds in this mixture (CA and CAPE) show antiviral, antimicrobial, antioxidant, anti-inflammatory and anticancer properties [3,4,5]. Our previous research showed that both compounds affect breast cancer cell viability, induce apoptosis, disrupt cell cycle progression and reduce cell migration [6,7]. The pro-apoptotic effects of CAPE have been demonstrated in ovarian cancer models while also showing synergy with paclitaxel [8]. The biological activity of CAPE exceeds CA, so we investigated its effects on tumour cells and their surrounding microenvironment components.

Ovarian cancer stands as the deadliest gynaecological cancer type which affects women. The combination of prevention initiatives and enhanced diagnostic methods has failed to decrease ovarian cancer mortality because patients receive late-stage diagnoses, and their tumours display different characteristics and tend to recur after treatment [9,10]. The disease exists as multiple distinct forms because it contains different histological types and genetic profiles and shows different clinical characteristics [11]. The PA-1 teratocarcinoma cell line serves as a model for studying this rare aggressive embryonal ovarian tumour [12]. The cell line functions as a research tool to investigate environmental elements which impact cancer cells and their surrounding tissue because it shows both pluripotent properties and superior differentiation capabilities [13]. The TME (tumour microenvironment) consists of immune cells, stromal fibroblasts, extracellular matrix components, adipocytes, pericytes, cytokines and metabolic signals, which work together to determine tumour progression. The initial antitumor response from cytotoxic T cells, macrophages and antigen-presenting cells gets overridden by immunosuppressive elements including regulatory T cells and M2-polarised macrophages, which enable tumour cells to evade immune detection and spread to distant sites [14,15]. The combination of connective tissue remodelling, hypoxia and angiogenesis in the stromal compartment enables tumours to survive and become more invasive [16,17]. Scientists must study all intricate system interactions to develop new treatment methods.

The digital twin modelling system allows researchers to analyse complex tumour systems through its powerful modelling capabilities. Researchers use computational models that link cell proliferation to immune cell entry, stromal tissue changes and hypoxia signalling to develop theoretical models, predict treatment effects and study untestable microenvironmental processes [18,19,20]. The research methods in gynaecological oncology help scientists create new treatments at lower experimental costs while improving their knowledge of biological systems. The study investigates CAPE effects on ovarian cancer microenvironment through experimental and computational methods. The study uses PA-1 cell biomarker data from laboratory tests together with digital twin modelling to discover the potential mechanisms which affect tumour–stromal–immune system interactions. Scientists can study ovarian cancer progression from natural compounds through this research method, which generates testable predictions for future biological studies.

In this work, we use the term “digital twin” to denote an in silico mechanistic counterpart of our in vitro ovarian cancer system—an ODE-based model focused specifically on tumour-microenvironment dynamics and parameterised using experimental and literature-derived values. This prototype is not a patient-specific clinical digital twin, but rather a computational reflection of the biological assays used in this study.

## 2. Materials and Methods

### 2.1. Experimental Cell Line

The study was conducted on the adherent PA-1 (CRL1572) teratocarcinoma cell line, derived from the ascites fluid of a 12-year-old Caucasian patient. Teratocarcinoma is a rare germ cell tumour with the ability to grow malignantly. It is characterised by the presence of various types of tissue derived from the three germ layers: ectoderm, mesoderm and endoderm. The cell line was supplied by Sigma Aldrich (catalogue number: 90013101, Warsaw, Poland). The PA-1 cell line was cultured according to the manufacturer’s guidelines using a Panasonic MCO-170AICUV incubator (Oizumi-Machi, Japan). A constant temperature of 37 °C and an atmosphere of 5% ${\mathrm{CO}}_{2}$ were maintained. The basic culture medium was Eagle’s Minimum Essential Medium (EMEM) supplied by Sigma Aldrich (catalogue number: 30-2003, Warsaw, Poland). The medium was supplemented with 5% foetal bovine serum (FBS) from PAA Laboratories (catalogue number: F4135, Pasich, Austria) and a mixture of antibiotics containing penicillin (100 IU/mL), streptomycin (100 μL/mL) and amphotericin B (250 μL/mL) were added to the medium. The cells were cultured in 25 ${\mathrm{cm}}^{2}$ bottles. All procedures were performed in an ALPINA BIO160 CYTO type A2 laminar flow cabinet using sterile disposable equipment. The medium was replaced every 48 h.

### 2.2. Tested Compound

Caffeic acid phenethyl ester (CAPE) with a purity of ≥97% was obtained from the supplier using HPLC (high-performance liquid chromatography)—Sigma Aldrich (compound catalogue number: C8221, Warsaw, Poland). Dilutions of the test compound (0–100 μM) were prepared by dissolving CAPE in DMSO (dimethyl sulfoxide). Previous studies have shown that the volume of DMSO used to prepare the solutions does not affect the viability and proliferation of the test cell line [8].

### 2.3. ELISA Tests

ELISA is an immunoenzymatic test based on the quantitative determination of the concentration of proteins contained in the test sample. The principle of the method is based on the formation of bonds between the antigen and the antibody, which are made visible in a colour reaction. The intensity of the colour depends on the number of antigen–antibody complexes formed. In the experiment, ELISA tests were used for the quantitative determination of decorin (Human Decorin ELISA Kit for Cell Culture Supernatants cat. no. RAB0140 Sigma Aldrich, Warsaw, Poland) and CA 72-4 (Human CD 72-4 ELISA for Cell Culture Supernatants; cat. no. RAB1308 Sigma-Aldrich) concentrations. Each of the determinations was performed in six replicates in two independent trials (n=12). To perform the assays, cell supernatants had to be prepared. For this purpose, the entire supernatant was first carefully removed from the culture vessels, and then the culture was gently rinsed with warm PBS. All supernatant was collected in centrifuge tubes and centrifuged at low speed (1500–2000 rpm) for 10 min to remove any cell debris and contaminants. The purified samples were transferred to clean eppendorf tubes and used immediately. The procedure for determining the concentration of decorin and CA 72.4 began with the preparation of reagents, i.e., bringing all parts of the kits to room temperature, diluting buffers and concentrates, and performing a series of standard dilutions in accordance with the instructions provided by the supplier. Next, 100 μL of standards/samples were added to ready-to-use plates coated with appropriate primary antibodies. The plates were incubated under foil for 2.5 h at room temperature on a shaker. After incubation, the material was removed from the plate and rinsed four times with 300 μL of washing buffer. Particular attention was paid to drying the entire contents of the well each time. After the last rinse, the plate was always drained on a paper towel. In the next stage of the assay, 100 μL of biotin-labelled secondary antibody was added to the wells. It was incubated for 1 h at room temperature using a shaker, and then rinsed again according to the procedure. After thorough drying, 100 μL of streptavidin solution was added to each well. It was incubated for 45 min at room temperature with gentle shaking. The plate was then rinsed again and MB One-Step Substrate Reagent was added for 30 min (incubation in the dark). Finally, 50 μL of STOP Solution was added to each well and the absorbance was read at a wavelength of 450 nm using an EL × 800 microplate reader (BioTek, Shoreline, WA, USA).

### 2.4. Statistical Analysis

The statistical analysis of data occurred independently for 24 h and 48 h incubation periods because the research used separate culture plates for each time point. The Shapiro–Wilk test evaluated data normality while the Levene test checked for equal variances between groups. Since both biomarkers exhibited unequal variances across CAPE concentrations (0–200 μM), the primary analysis of concentration effects used Welch’s heteroscedastic one-way ANOVA, which does not assume equal variances. When global significance was detected, Games–Howell post hoc testing was applied for pairwise comparisons, as it is appropriate for unequal variances and unequal standard deviations. For completeness and methodological robustness, a non-parametric analysis was also performed. The Kruskal–Wallis test, followed by Dunn’s post hoc test with Holm correction, was used as a conservative, distribution-free alternative to confirm dose-dependent differences. Each condition included 8 biological measurements, derived from three independent experiments, each containing multiple technical replicates. Statistical significance was set at *p* < 0.05, with significance levels denoted as *p* < 0.05 (*), *p* < 0.01 (**), and *p* < 0.001 (***).

### 2.5. Digital Modelling

The digital twin framework presented here integrates our in vitro CAPE response data with a TME-focused system of ODEs, enabling quantitative simulation of angiogenic and cytotoxic signalling. The model does not aim for patient-level personalisation; instead, it functions as an experimentally anchored computational surrogate to explore mechanistic hypotheses.

The simulation of ovarian cancer microenvironment required ordinary differential equations (ODEs) to model its complex interactions. The mathematical system allows us to combine cellular processes (e.g., cancer proliferation and apoptosis) with immune system elements ($CD8^{+}$ T cells, Tregs and macrophage polarisation) and microenvironmental components (e.g., hypoxia, angiogenesis and extracellular matrix remodelling).

Wherever below variables are used, the meanings are as follows: *T*—tumour cells; *E*—$CD8^{+}$ effectors; *R*—Tregs; Th—Th1; APC—activated dendritic cells; $M_{1}$/$M_{2}$—macrophage phenotypes; *F*—Cancer-Associated Fibroblasts (CAFs); *S*— extracellular matrix (ECM) stiffness; MMP—matrix proteases; $O_{2}$—oxygen; *G*—glucose; *H*—hypoxia/hypoxia-inducible factor (HIF) activity; *V*—VEGF; *A*—functional vessels; *L*—lactate; *C*—immunosuppressive cytokines (TGF-β/IL-10 lumped); CX—CXCL12; PD1, PDL1—checkpoint receptors; CpxPD-1:PD-L1 complexes; *N*—NF-κβ; *P*—p53; *D*—drug effect input (e.g., CAPE); *Q*—adipocyte/CAA activity; $F_{a}$—free fatty acids; Lep—leptin; Adn—adiponectin; IL6—interleukin-6; CCL2—monocyte chemoattractant protein-1.

Tumours expand with nutrient-limited growth and are curtailed by Cytotoxic T lymphocytes (CTLs) and macrophages *M1* while supported by *M2* and stressed by hypoxia, consistent with contemporary TME biology [14].

(1) $$\frac{dT}{dt}=r_{T}\;T\;\left(1-\frac{T}{K_{T}}\right)-\frac{k_{E}\;E\;T}{T+K_{E}}-\frac{k_{M1}\;M_{1}\;T}{T+K_{M1}}+k_{M2}\;M_{2}\;T-k_{\mathrm{hyp}}\;\frac{H}{K_{H}+H}\;T$$

Effector T cells are promoted by APC/IL-2 priming and chemokine/vessel-supported trafficking but are curtailed by Tregs, stiff ECM and checkpoint engagement [21,22].

(2) $$\begin{array}{c} \frac{dE}{dt}=s_{E}+k_{act,E}\;\frac{APC}{K_{APC}+APC}\;\frac{IL2}{K_{IL2}+IL2}+\chi_{A}\;\frac{A}{K_{AE}+A}\\ +\chi_{CX}\;\frac{CX}{K_{CX}+CX}-\mu_{R}\;R\;E-\mu_{S}\;S\;E-k_{exh}\;\frac{Cpx}{K_{Cpx}+Cpx}\;E-\delta_{E}\;E \end{array}$$

We combined Equation (1) for tumour growth with Equation (2) for effector T-cell dynamics to capture the reciprocal interactions between cancer cells and immune surveillance, allowing simulation of how CAPE simultaneously suppresses tumour proliferation and enhances immune activation (3):

(3) $$\begin{array}{c} \frac{dT}{dt}=rT\left(1-\frac{T}{K_{T}}\right)-kET\;\left(1+\phi \;f(CAPE)\right)\\ \frac{dE}{dt}=s_{E}+\frac{p_{E}T}{T+K_{E}}\;\left(1+\eta f\left(CAPE\right)\right)-d_{E}E-\alpha_{Treg}RE \end{array}$$

where assumptions are

- logistic growth term: $rT(1-T/K_{T})$;
- effector killing proportional to E·T;
- CAPE enhances effector killing efficiency ϕf(CAPE);
- CAPE enhances effector proliferation ηf(CAPE);
- activation from tumour antigen load item natural death + suppression from Tregs.

Tregs accumulate in response to TGF-β/IL-10 signals and are further stabilised by lactate-rich, immunosuppressive metabolism [23]. Lactate and suppressive cytokines bias macrophages toward *M2*, whereas effector/interferon (IFN-γ) cues favour M1 polarisation [24].

(4) $$\begin{array}{c} \frac{dR}{dt}=s_{R}+\alpha_{R}\;C+\beta_{R}\;L-\delta_{R}\;R. \end{array}$$

where *R* denotes Regulatory T cell (Treg) population—immunosuppressive cells that suppress effector T cell activity; *C* denotes suppressive cytokines (e.g., TGF-β, IL-10)—signals that promote Treg activation and expansion; *L* denotes lactate concentration—a metabolic by-product of tumour glycolysis, which stabilises Tregs in hypoxic/immunosuppressive niches; $s_{R}$ denotes baseline source rate of Tregs (homeostatic recruitment from periphery); $\alpha_{R}$ denotes strength of cytokine-driven Treg induction (sensitivity to suppressive cytokines like TGF-β); and $\beta_{R}$ denotes strength of lactate-driven Treg stabilisation (how much metabolic by-products support Treg expansion).

Lactate and suppressive cytokines bias macrophages toward *M2*, whereas effector/interferon (IFN-γ) cues favour *M1* polarisation [24].

(5) $$\begin{array}{c} \frac{dM_{1}}{dt}=s_{M1}+\alpha_{M1}\;E-\beta_{L1}\;L\;M_{1}-\delta_{M1}\;M_{1} \end{array}$$

(6) $$\begin{array}{c} \frac{dM_{2}}{dt}=s_{M2}+\beta_{L2}\;L+\gamma_{C2}\;C-\alpha_{E2}\;E\;M_{2}-\delta_{M2}\;M_{2} \end{array}$$

Cancer-associated fibroblasts (CAFs) expand in response to tumour and TGF-β, orchestrating stromal remodelling in ovarian cancer [25].

(7) $$\begin{array}{c} \frac{dF}{dt}=s_{F}+\alpha_{F}T+\gamma_{F}C-\phi_{F}F-\kappa_{F}\;f\left(D\right)\;F \end{array}$$

where f(D) is the Hill function of CAPE dose, and $\kappa_{F}$ the strength of CAPE suppression on CAFs.

CAFs and *M2* increase ECM stiffness which excludes T cells; proteases remodel and soften the matrix [22].

(8) $$\begin{array}{c} \frac{dS}{dt}=\rho_{S}\;F+\lambda_{S}\;M_{2}-\delta_{S}\;S-\delta_{S}^{\prime }\;MMP\;S \end{array}$$

(9) $$\begin{array}{c} \frac{dMMP}{dt}=\alpha_{MMP,T}\;T+\alpha_{MMP,F}\;F-\mu_{MMP}\;MMP \end{array}$$

Hypoxia (low $O_{2}$) stabilises HIF (Hypoxia-Inducible Factor) which reprograms metabolism and promotes angiogenic signalling [26].

(10) $$\begin{array}{c} \frac{dH}{dt}=\alpha_{H}\;\frac{1}{1+O_{2}/K_{O_{2}}}\;\frac{T}{K_{TH}+T}-\delta_{H}\;H \end{array}$$

where H denotes HIF-1α activity (proxy for hypoxia signalling); $O_{2}$ denotes oxygen concentration; *T* denotes tumour cells (driving demand and hypoxia); $K_{O_{2}}$, $K_{TH}$ denote saturation constants; $\alpha_{H}$ denotes production rate of HIF driven by hypoxia and tumour size; and $\delta_{H}$ denotes degradation rate of HIF.

HIF-induced VEGF drives vessel formation that partially relieves hypoxia but remains abnormal [26].

(11) $$\begin{array}{c} \frac{dV}{dt}=\alpha_{V}\;H-\delta_{V}\;V \end{array}$$

(12) $$\begin{array}{c} \frac{dA}{dt}=\alpha_{A}\;\frac{V}{K_{VA}+V}\left(1-\frac{A}{A_{max}}\right)-\delta_{A}\;A \end{array}$$

where VEGF production (*V*) is induced by HIF-1α (*H*) and degraded at rate $\delta_{V}$; and abnormal vessel density (*A*) grows in response to VEGF with saturation $K_{VA}$ and a carrying capacity $A_{\max}$, but is degraded at rate $\delta_{A}$.

All simulations and numerical calculations were performed using MATLAB R2025a (MathWorks, Natick, MA, USA).

### 2.6. Simulation Parameters

The parameters used in the ODE-based digital twin were implemented by combining values from our MATLAB simulations with biologically plausible ranges reported in established tumour–immune models. Core terms regulating tumour growth, T-cell activation, and cytotoxicity were taken from well-validated literature sources, while the CAPE-dependent suppression term (δ) was calibrated phenomenologically to reflect the dose–response observed in our in vitro assays. This approach ensures that the model remains mechanistically interpretable while capturing the experimentally observed effects of CAPE. A limitation of this method is that several parameters rely on literature estimates or normalisation rather than direct experimental measurement, and the CAPE effect term reflects empirical mapping rather than fully mechanistic kinetics (Table 1, Table 2, Table 3, Table 4, Table 5 and Table 6).

## 3. Results

### 3.1. Experimental Results

**Figure 1:**
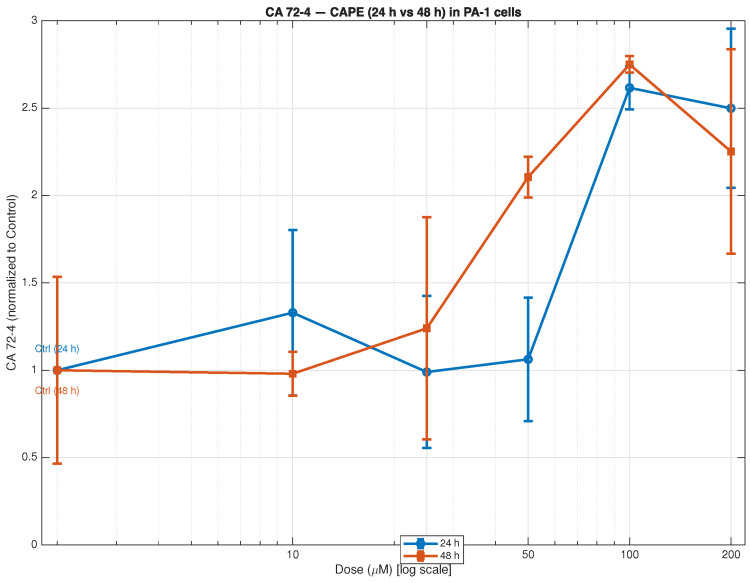
CA 72-4 expression in PA-1 ovarian teratocarcinoma cells following CAPE treatment (24 h and 48 h). PA-1 cells were treated with increasing concentrations of caffeic acid phenethyl ester (CAPE; 0–200 μM) for 24 h (blue) and 48 h (orange). CA 72-4 levels were quantified by ELISA and normalised to untreated controls (Control = 1). Data represent mean ± SD from eight independent experiments. A logarithmic dose scale was applied to highlight concentration-dependent trends. A time- and dose-dependent increase in CA 72-4 was observed, with the highest CAPE concentrations (100–200 μM) producing the strongest responses at 48 h. These results indicate that CAPE modulates tumour-associated glycoprotein secretion in PA-1 cells, reflecting stress-related and apoptotic responses. Statistical analysis is shown in Appendix A Table A1 and Table A2.

The research measured CA 72-4 levels in PA-1 ovarian teratocarcinoma cells following CAPE treatment at 10 to 200 μM concentrations for 24 and 48 h (Figure 1). The CA 72-4 expression levels demonstrated a dose-dependent pattern which increased at intermediate and higher CAPE concentrations with the most significant rise observed at 100 μM and a decrease at 200 μM. The levels of CA 72-4 remained near control values at 24 h when cells received CAPE concentrations up to 50 μM but showed progressive elevation starting at 25 μM during 48 h, reaching its peak at 100 μM (2.8 times higher than control).

**Figure 2:**
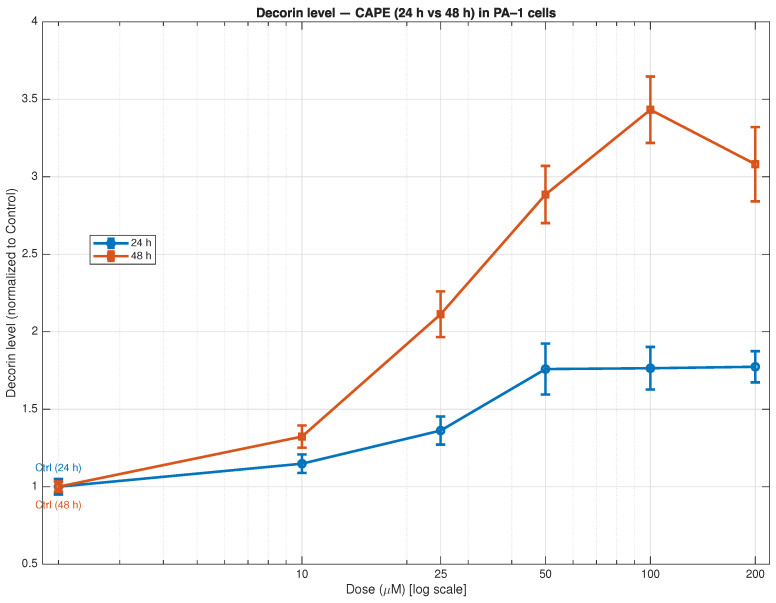
Effect of caffeic acid phenethyl ester (CAPE) on decorin expression in PA-1 ovarian teratocarcinoma cells at 24 h and 48 h. Decorin levels were measured by ELISA and normalised to untreated control (set as 1). CAPE induced a dose- and time-dependent increase in decorin expression, with a stronger effect observed after 48 h of treatment. At 24 h, decorin levels rose moderately at ≥25 μM, while at 48 h they increased sharply, peaking at 100 μM before a slight decline at 200 μM. Data represent mean ± SD from eight independent experiments). Statistical analysis is shown in Appendix A Table A1 and Table A2.

The expression of decorin in PA-1 ovarian teratocarcinoma cells increased substantially through CAPE treatment at different concentrations and time points (Figure 2). The decorin levels increased moderately at 24 h starting from 25 μM before reaching a plateau between 50 and 200 μM to achieve 1.8 times the control levels. The 48-h CAPE treatment produced a stronger and continuous decorin increase which started at 10 μM and reached its maximum at 100 μM while exceeding control levels by 3.5 times. The decorin levels decreased slightly at 200 μM because of toxic effects that occur when cells experience high concentrations of the compound.

### 3.2. Modelling Results

The combined model of tumour dynamics and effector T cell expansion (Equation (3)) demonstrates how CAPE affects cancer cells and immune cells within the ovarian cancer microenvironment. The tumour equation demonstrated that PA-1 cell numbers decreased proportionally to the concentration of CAPE. The tumour growth rates decreased with each increase in CAPE concentration from 10 to 100 μM until the highest concentration of 100 increases with rising CAPE concentration in a dose-dependent manner.

The model predicts that changes in $CD8^{+}$ T cell numbers produce three main effects:

1. The death of tumour cells caused by CAPE treatment leads to increased availability of antigens for $CD8^{+}$ T cells to recognise which results in their population growth.
2. The immune system receives direct activation from CAPE which leads to increased cell proliferation and survival of effector cells. The reduction in tumour-mediated immunosuppression enables $CD8^{+}$ cells to expand better because regulatory suppression becomes less effective.
3. The immune system transitions from tumour-mediated suppression to activation through CAPE treatment which enables $CD8^{+}$ cells to multiply and fight cancer more effectively.

**Figure 3:**
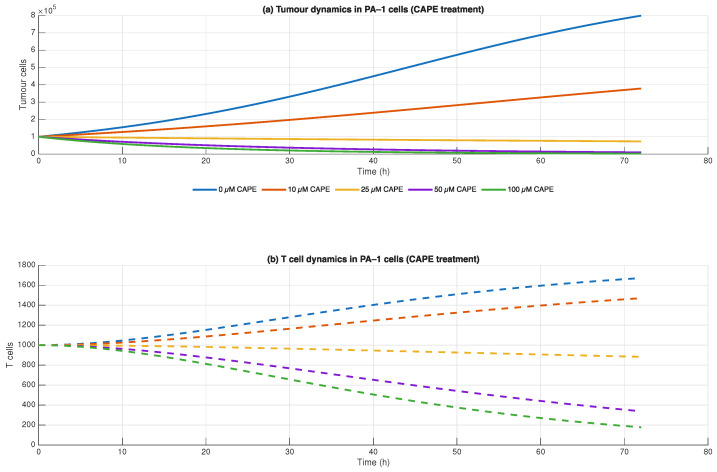
Simulated effects of CAPE (10–100 μM) on PA-1 ovarian cancer cells. Solid lines represent tumour cell populations, while dashed lines represent T cell dynamics. Simulations were based on coupled ODE models (Equation (3)).

The research demonstrates that CAPE both stops tumour growth directly and strengthens the body’s immune response against cancer cells. The dual mechanism of action makes CAPE suitable for use as both a cytotoxic agent and an immunomodulatory agent in ovarian cancer treatment. The results of modelling are shown in the Figure 3.

Equations (4)–(6) were used to model the effects of CAPE on regulatory T cells (Tregs) and macrophage polarisation within the tumour microenvironment of PA-1 ovarian cancer cells. As described in the model, Tregs accumulate in response to TGF-β/IL-10 signalling and lactate-rich metabolism, while macrophage polarisation depends on suppressive cues (M2) versus effector/IFNγ inputs (M1).

**Figure 4:**
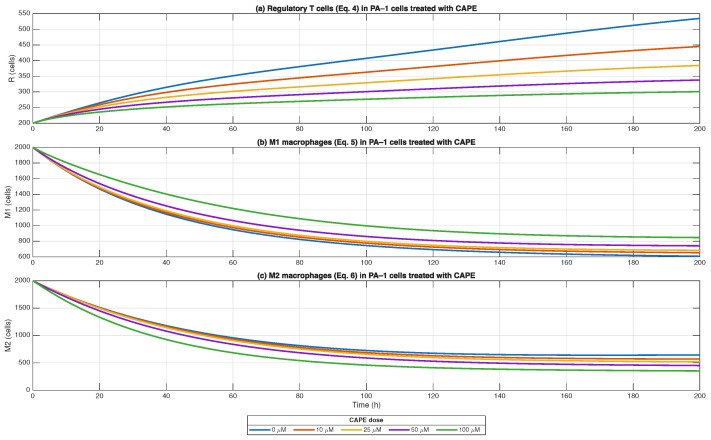
Simulation results of immunoregulation block showing the effects of CAPE on regulatory T cells, *M1* macrophages, and *M2* macrophages, in PA-1 ovarian cancer cells. Dose–response panels (0, 10, 25, 50, 100 μM CAPE) were simulated over 200 h. CAPE reduced Treg accumulation, enhanced *M1* polarisation, and suppressed *M2* levels, with ca. +50% *M1* and ca. −50% *M2* relative to control at 72 h under 100 μM CAPE, illustrating CAPE’s role in reprogramming the tumour microenvironment toward anti-tumour immunity.

The simulations showed that CAPE treatment induced a dose- and time-dependent reprogramming of these populations. At baseline (0 μM), Tregs and *M2* macrophages remained dominant, supporting an immunosuppressive state, while *M1* macrophages were limited. With CAPE exposure, Treg levels progressively decreased, especially at doses ≥ 50 μM, consistent with reduced TGF-β/IL-10 signalling. In parallel, *M1* macrophages increased with dose, reaching around 50% above control at 72 h under 100 μM CAPE, while *M2* macrophages declined to ca. 50% of control under the same conditions. These findings highlight CAPE’s ability to attenuate immunosuppressive signals and shift macrophage balance from *M2* to *M1*, thereby supporting effector T-cell function and strengthening the anti-tumour immune response within the simulated ovarian cancer microenvironment (Figure 4).

**Figure 5:**
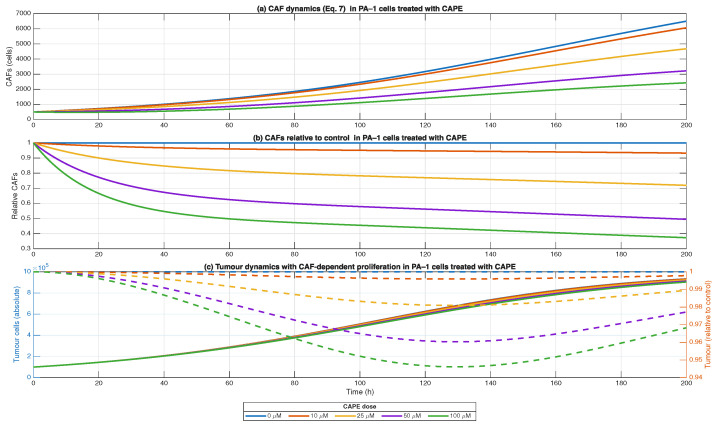
Cancer-associated fibroblasts (CAFs) and their feedback on tumour growth under CAPE treatment. Dynamics of CAF population based on Equation (7) in PA-1 ovarian cancer cells exposed to increasing CAPE doses (0, 10, 25, 50, 100 μM). CAPE caused a dose-dependent suppression of CAF expansion, reaching ∼50% reduction at 72 h with 100 μM. Tumour cell dynamics incorporating CAF/TGF-β feedback, where CAF abundance modulates effective tumour proliferation rate. Reduced CAF levels under CAPE treatment slowed tumour expansion compared to untreated control. Overlay of tumour trajectories under different CAPE doses, illustrating the indirect effect of CAF suppression on tumour growth. Data represent simulations of ODE system (3)–(7) with calibrated CAPE feedback parameters. Solid lines represent CAF dynamics (left Y-axis), showing the dose-dependent suppression of fibroblast activity under CAPE treatment, while dashed lines represent tumour cell dynamics (right Y-axis) under varying CAPE doses, capturing how changes in CAF abundance feed back via TGF-β signalling to modulate effective tumour growth.

The immunosuppressive cytokines (TGF-β, IL-10) according to the equation model shows that cancer-associated fibroblasts (CAFs) grow based on tumour size (Equation (7)). The CAFs showed continuous growth under normal conditions because they play a role in stromal tissue reshaping and tumour cell support. The application of CAPE treatment resulted in dose-dependent CAF reduction which reached a ∼50% decrease at 100 μM concentration after 72 h compared to untreated controls Figure 5.

The model received an additional feedback mechanism that showed that CAF numbers affect the tumour cells’ ability to proliferate. The model demonstrates how CAFs support tumour growth through their secretion of growth factors and their TGF-β-dependent extracellular matrix remodelling activities. CAF suppression by CAPE resulted in decreased tumour growth rates. The strength of CAF suppression by CAPE increased with higher drug concentrations, which resulted in more pronounced inhibition of the tumour growth.

The study demonstrates that CAPE works against tumour progression by controlling stromal cell growth particularly through CAF reduction and TGF-β signalling pathway inhibition (Figure 3).

**Figure 6:**
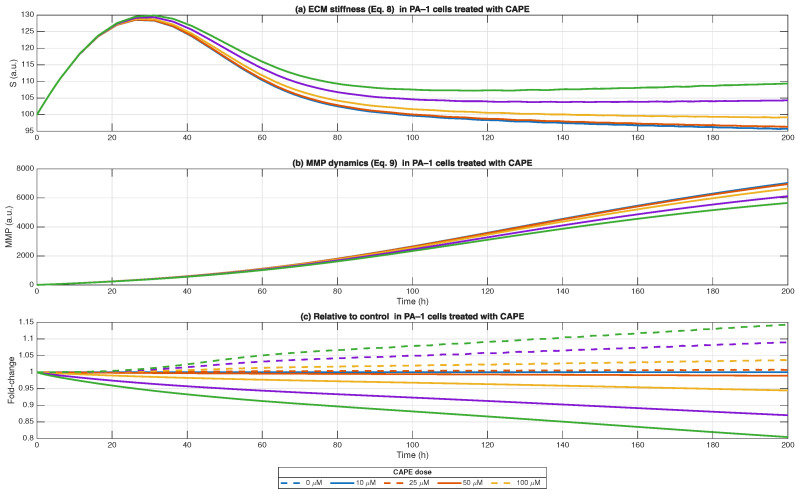
ECM stiffness and MMP dynamics under CAPE treatment in PA-1 cells. ECM stiffness (Equation (8)) over time in response to CAPE (0–100 μM). Higher doses reduce stiffness after an initial peak, reflecting impaired stromal support. MMP dynamics (Equation (9)), showing sustained accumulation of matrix metalloproteinases, with higher CAPE doses producing modest suppression. Relative fold-change compared to control. Solid lines indicate ECM stiffness, while dashed lines indicate MMP levels. CAPE progressively decreases ECM stiffness and moderately limits MMP-driven remodelling, thereby reducing the structural support available for tumour growth.

We studied tumour microenvironment components by combining cancer-associated fibroblasts (CAFs) and *M2* macrophage effects on extracellular matrix (ECM) stiffness (Equation (8)) with matrix metalloproteinase (MMP) activity (Equation (9)), all in Figure 4. The model shows that CAFs and *M2* macrophages made the ECM more rigid which blocks T-cell entry, but MMPs from tumour cells and CAFs break down the matrix structure. Moreover, ECM stiffness was increased at first because of CAF and *M2* cell growth but then decreased steadily because MMP degradation activities became dominant. CAPE treatment caused a dose-dependent decrease in tissue stiffness when compared to untreated samples (Figure 4c dashed lines). The highest doses of CAPE (≥50 μM) produced the most significant effect on matrix softening because they reduced stiffness at a faster rate. The time-dependent MMP accumulation showed an increase in absolute values throughout all experimental conditions (Figure 4b). The relative MMP activity measurements (Figure 6 solid lines) demonstrated that CAPE treatment increased MMP activity in a concentration-dependent fashion which matched the observed decrease in ECM stiffness. The findings show that CAPE reduced stromal reinforcement through CAF and *M2* signal suppression while creating an environment with less immunosuppressive properties. The computational model indicates that CAPE treatment decreased physical barriers in the ECM while promoting matrix degradation which could improve immune cell penetration and anti-tumour effects in ovarian cancer. When integrated with the immunoregulatory module (Equations (4)–(6)), these findings highlight a mechanistic link: CAPE-driven macrophage re-polarisation (M2→M1) reduces the pro-stiffness influence of *M2* macrophages while favouring pro-degradative pathways. The dual mechanism of CAPE action includes suppression of *M2* macrophages and promotion of MMP-dependent tissue remodelling, which enables T cells to penetrate ovarian cancer microenvironments and perform their functions effectively.

**Figure 7:**
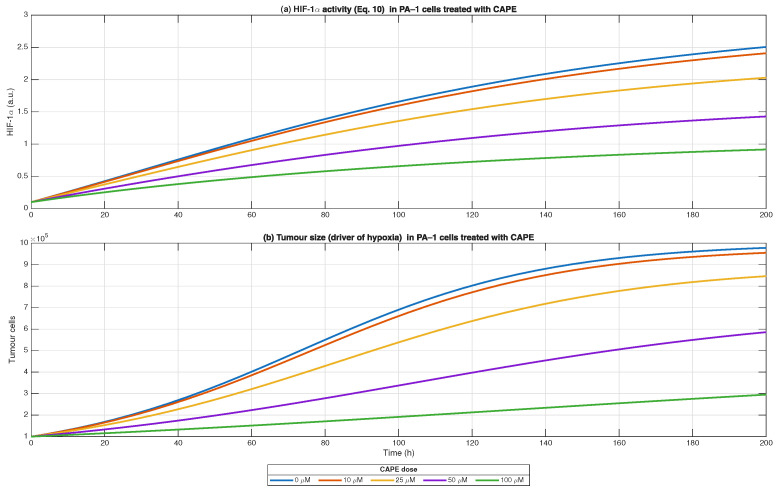
Simulation of hypoxia–HIF dynamics in PA-1 ovarian cancer cells treated with CAPE. HIF-1α activity increased over time as tumour growth progressed, reflecting hypoxia-induced stabilisation of HIF-1α. CAPE treatment attenuated HIF-1α accumulation in a dose-dependent manner by enhancing oxygen availability and degradation while suppressing induction. Tumour growth dynamics (logistic model) under increasing CAPE concentrations (0–100 μM) demonstrate reduced expansion compared to control. Solid lines represent simulations for individual CAPE doses; legend indicates concentration. Results illustrate the dual impact of CAPE on tumour size and hypoxia-driven HIF-1α signalling within the microenvironment.

The simulation of hypoxia–HIF dynamics (Equation (10) showed that HIF-1α accumulation in PA-1 cells directly correlated with tumour growth Figure 7. The activity of HIF-1α in untreated control cells grew progressively as the tumour size increased because the expanding cell population experienced oxygen deficiency. The suppression of HIF-1α accumulation increased with CAPE concentration where the highest doses (50–100 μM) produced the most significant reduction. The treatment of CAPE produced two separate effects which led to its outcome: (i) the decrease in tumour mass reduced hypoxic conditions and (ii) the treatment improved HIF-1α breakdown and oxygen usage efficiency. The treatment with CAPE transformed the tumour environment from a hypoxia-promoting pro-angiogenic state into a condition with decreased HIF signalling which could enhance drug delivery and immune cell entry.

The HIF-1α dynamics described in Equation (10) served as the basis for modelling VEGF production through Equation (11) as a response to HIF stabilisation during hypoxic conditions. The accumulation of VEGF through tumour-driven hypoxia signalling occurred steadily as HIF activity increased in untreated control samples. The highest concentration of 100 μM CAPE showed the most effective reduction in VEGF production, while lower concentrations of CAPE produced less suppression.

**Figure 8:**
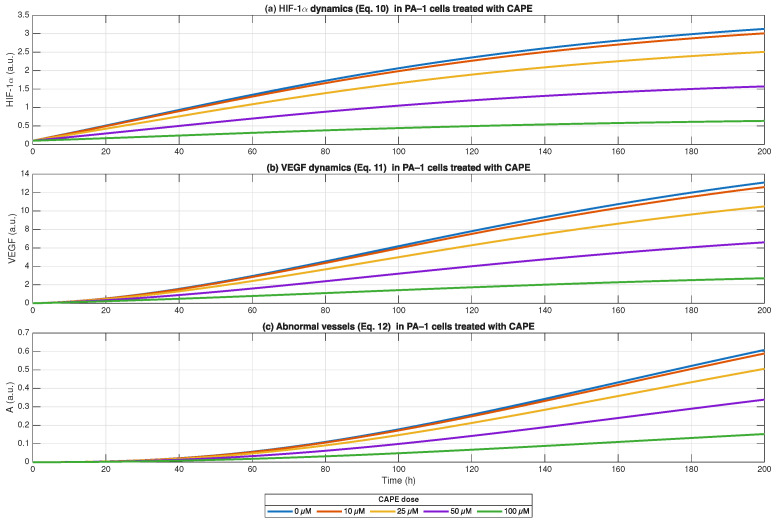
Hypoxia–VEGF–angiogenesis dynamics in PA-1 cells treated with CAPE. HIF-1α dynamics show progressive accumulation under control conditions, with CAPE reducing HIF-1α activity in a dose-dependent manner. VEGF production closely follows HIF-1α trends, with control cells showing continuous increase, whereas CAPE treatment suppressed VEGF signalling proportionally to dose. Abnormal vessel formation rises under hypoxia-induced VEGF drive in untreated cells but is significantly attenuated by CAPE, particularly at 50–100 μM. Together, these results indicate that CAPE shifts the tumour microenvironment away from hypoxia-driven angiogenesis.

The relationship between VEGF signalling and abnormal angiogenic structure growth is established through Equation (12) and shown in Figure 8. The simulation results showed that abnormal vasculature (A) approached its maximum value because VEGF continued to stimulate angiogenesis throughout the simulation period. The rate of vessel formation decreased with each dose increase of CAPE until the 100 μM concentration completely blocked abnormal angiogenic growth.

The Equations (10)–(12) show that CAPE disrupts tumour progression by blocking HIF-1α-driven VEGF production and stopping the growth of pathological blood vessels which are essential for tumour development.

## 4. Discussion

The tumour niche is a complex system of various extracellular matrix components, cells and the cytokines and chemokines they produce, which create an environment that promotes tumour growth. A review of the literature shows that, in addition to cancer cells, the microenvironment also includes leukocytes, connective tissue cells (including fibroblasts and adipocytes) and elements of the vessel wall. Ovarian cancer cells have the ability to shed certain surface antigens, overexpress proteins that inhibit the immune response (e.g., PD-L1) and recruit immunosuppressive cells (Treg, *M2* macrophages) to their niche [64]. The specific conditions prevailing in the primary tumour site promote the development of hypoxia and metabolic changes that accelerate proliferation but may also limit drug access and treatment efficacy. It therefore seems particularly important to understand the local processes accompanying tumour formation and to develop precise therapeutic targets [65].

CA 72-4 (cancer antigen 72.4) is a high-molecular-weight mucin-like glycoprotein (TAG-72, tumour-associated glycoprotein-72), which, under physiological conditions, occurs in small amounts on the cell membrane of the gastrointestinal tract mucosa (mainly the stomach) and endometrium. Increased expression of the antigen has been associated with the malignant transformation of many cancers, including serous and mucinous ovarian cancer [66]. However, the diagnostic sensitivity of CA72.4 in detecting ovarian cancer is approximately 40–60% and is lowest in stage I of the disease, while its specificity of over 90% allows the source of tumour growth to be clearly identified. It is therefore suggested that CA 72.4 should be measured simultaneously with CA125 and HE4 and that this marker should be used for predicting the course of the disease, monitoring therapy and detecting recurrences. It has not been investigated yet whether CA72.4 concentration can be correlated with teratocarcinoma [67,68].

The delayed yet stronger upregulation at 48 h indicates that CAPE creates prolonged cellular stress which leads to increased apoptotic signalling that results in elevated secretion of tumour-associated glycoproteins like CA 72-4 when cells undergo apoptosis. The study shows that CAPE controls tumour cell secretion patterns through time-dependent and concentration-based mechanisms which might result from apoptotic cell death and modified tumour cell membrane glycoproteins.

The observed initial reduction in CA 72-4 expression suggests that CAPE interrupts cell surface mucin expression and potentially the secretory phenotype of tumour cells, consistent with its known pro-apoptotic and antiproliferative effects [69]. Hence, the CAPE-driven decline in CA 72-4 may reflect a shift of tumour cells toward a less aggressive phenotype and increased susceptibility to apoptotic signals. In other tumour models, CAPE has been shown to inhibit NF−κβ activation and enhance p53-mediated apoptosis, mechanisms that would suppress mucin-producing and survival-promoting gene expression [70,71].

The down-modulation of CA 72-4 aligns with CAPE’s ability to limit tumour cell viability and secretory output. Moreover, since elevated CA 72-4 may also signal enhanced mucin barrier formation and immune escape, its reduction suggests enhanced exposure of tumour antigens and improved immune recognition. This connects with our immune-microenvironment modelling showing increased effector-cell activation under CAPE.

However, it is worth noting that CA 72-4 by itself has limitations in sensitivity and specificity, especially in healthy populations. For example, an asymptomatic cohort with raised CA 72-4 did not show increased malignancy risk at long-term follow-up [72]. Thus, the marker’s prognostic value may depend heavily on context and underlying tumour biology. In the case of CAPE-treated ovarian models, CA 72-4 decline likely captures enhanced apoptotic and secretory shutdown rather than purely tumour burden reduction.

While CA72-4 is widely used as a tumour-associated antigen and often correlates with advanced disease stage and metastasis in clinical settings, its elevation in in vitro monoculture settings should be interpreted with caution. CA72-4 is a high-molecular-weight mucin-like glycoprotein that can be shed or secreted from tumour cells—a process that may be enhanced by cellular stress, membrane perturbation, or apoptotic signalling, rather than by enhanced proliferation or invasiveness [73]. Indeed, recent clinical and epidemiological evidence shows that elevated CA72-4 levels are sometimes observed in non-malignant conditions or even in asymptomatic healthy individuals with no detectable cancer on follow-up. For example, in a prospective study of healthy individuals with raised CA72-4, the long-term incidence of malignancy was low and not significantly different from the general population [74]. Similarly, CA72-4 levels have been reported to rise transiently in inflammatory conditions such as gout flares, suggesting that inflammation or metabolic stress can trigger antigen shedding [75]. In light of these observations, the dose-dependent increase in CA72-4 observed in our PA-1 in vitro model after CAPE exposure is more plausibly interpreted as stress-induced shedding or altered glycoprotein processing, possibly related to CAPE’s cytotoxic or membrane-perturbing effects—rather than as a sign of increased tumour aggressiveness or metastatic potential. This interpretation aligns with the concurrent reduction in cell viability and impairment of proliferative capacity under CAPE treatment observed in our experiments. Therefore, while CA72-4 remains a useful clinical biomarker in vivo, in vitro elevations—especially under cytotoxic or stress-inducing treatments—should be viewed as biochemical indicators of cellular distress, not as direct evidence of enhanced malignant potential. We recommend interpreting CA72-4 changes in cell culture with caution and using complementary functional assays when drawing conclusions about aggressiveness or metastatic risk.

Taken together, our findings extend CAPE’s anticancer profile by showing that it reduces not only intracellular survival signalling and proliferation but also tumour-associated surface/secretory markers like CA 72-4. This integrated effect highlights CAPE’s potential as an adjuvant targeting both tumour phenotype and microenvironmental-immune interactions. Future work should explore whether the CA 72-4 reduction correlates with improved immune infiltration and patient-relevant outcomes in vivo.

The results of CA72-4 analysis in our tumour microenvironment model highlight the role of TME components—including *M2* macrophages, tumour-associated fibroblasts (CAFs) and the extracellular matrix—in ovarian cancer progression. As recent reviews show, the tumour microenvironment in ovarian cancer includes not only cancer cells, but also immune cells, stromal cells, the extracellular matrix and physicochemical factors, which affect both cancer cell survival and therapeutic response [76]. In the context of CA72-4, which reflects the expression of mucin-type glycoprotein (TAG-72) in ovarian tumours and is less susceptible to hormonal interference than classical markers [77], its elevated level may be a signal of stromal-matrix and immunomodulatory mechanisms promoting tumour progression. In particular, CAFs and *M2* macrophages secrete TGF-β and IL-10, which promote immunosuppression, remodel the ECM, and support tumour malignancy [78]. Our data suggest that an increase in CA72-4 may reflect the activation of these TME components, confirming that this marker not only monitors tumour mass but also its microenvironmental interactions. Therefore, CA72-4 may serve as an intermediary linking tumour biomarkers with microenvironmental status assessment and indicate the targeting of therapy not only to tumour cells but also to their niche. The rationale for including the aforementioned parameters was to ensure that the digital twin faithfully reproduces the functional behaviour of the tumour microenvironment.

Decorin is a component of the extracellular matrix classified as a small leucine-rich proteoglycan (SLRP). It consists of a protein core and a single chain of chondroitin sulphate or dermatan sulphate and is produced mainly by fibroblasts, myofibroblasts and smooth muscle myocytes (also found in the walls of blood vessels) [79]. The highest tissue concentrations of decorin are found in parenchymal organs, including the ovary. It has also been shown that decorin levels in the stroma of cancerous tumours are higher than in physiological tissues.

The results show CAPE triggers decorin expression to increase at different concentrations and time points which matches the activation of extracellular matrix (ECM)-modulating pathways. The elevated decorin levels indicate stronger stromal structure maintenance and tumour-controlling environmental changes that match decorin’s proven ability to block TGF-β signalling, receptor tyrosine kinase activity and angiogenesis in a tumour stroma.

The study shows that PA-1 ovarian teratocarcinoma cells express decorin at higher levels through time-dependent and dose-dependent mechanisms when treated with CAPE. The extracellular matrix (ECM) contains decorin as a small leucine-rich proteoglycan (SLRP) which functions as a multi-functional component that blocks receptor tyrosine kinases (RTKs) and binds TGF-β and controls collagen formation and influences immune cell responses [80]. The presence of decorin in tumour stroma is commonly seen as a protective factor which indicates a less aggressive tumour environment with reduced fibrosis [81]. The TME (tumour microenvironment) shows evidence of restored ECM structural controls through decorin upregulation which CAPE triggers because it blocks TGF-β activation of fibroblasts and decreases collagen accumulation and matrix stiffness [82].The desmoplastic stroma and elevated ECM stiffness in ovarian cancer create conditions that enable immune evasion and reduce drug delivery effectiveness which results in worse patient outcomes. The increased decorin levels indicate positive changes in the TME which create an environment that becomes less conducive to tumour expansion and metastasis. The integrin-mediated adhesion and mechanotransduction functions of decorin help decrease the development of invasive tumour cell phenotypes [83].

The combination of decreased ECM signalling and restricted RTK activation through elevated decorin levels leads to reduced tumour cell growth and adhesion and enhanced immune cell entry into the less rigid microenvironment. The research supports the need to focus on stromal and matrix components as therapeutic targets because conventional tumour cell-focused cytotoxic approaches prove insufficient for effective treatment [22].

Our research shows that CAPE treatment of PA-1 cells leads to decorin expression increase which demonstrates stromal modification and supports a therapeutic approach that transforms the TME from fibrotic to immune-accessible, drug-permeable and less invasive.

Decorin is a protein that not only cross-links collagen fibres, giving shape to the intercellular matrix, but also plays an important role in regulating cell growth and proliferation. Under physiological conditions, decorin shows affinity for growth factors such as TGF-β, IGF-I, HGF, and PDGF, inhibiting their activity, limiting cell division and connective tissue remodelling. By binding to VEGF, it can also influence the formation of new blood vessels [84]. The binding of decorin to the EGF receptor causes its internalisation and degradation, which prevents the action of mitosis-stimulating factors. Moreover, decorin is considered to be a physiological brake on excessive fibrosis and uncontrolled cell division [83]. It has been shown that this protein has a direct effect on the expression of $p21^{CIP1/WAF1}$ genes, which are cell cycle inhibitors that arrest cells in the G1 phase and can activate executive caspases (−3 and −8) [85].

Literature reports have demonstrated that reduced concentrations of decorin measured in ovarian cancer cells and tumour stroma correlated with histological malignancy, higher clinical stage (according to FIGO) and risk of metastasis [86]. It is therefore proposed to assess the level of this protein as a prognostic marker for estimating the prognosis of patients. Since decorin can inhibit the proliferation and migration of cancer cells, induce apoptosis in them, and block the formation of new blood vessels, there are also studies evaluating the effectiveness of therapy using recombinant decorin. A study showed that the administration of exogenous decorin to ovarian cancer cell cultures reduced their proliferation and ability to colonise [87].

Our integrated digital twin framework combining immune regulation, CAF dynamics, and stromal remodelling provides a systems view of how CAPE may reshape the tumour microenvironment. The simulations predict that CAPE suppresses CAF expansion and *M2* macrophage pro-stiffness signalling, thereby weakening ECM rigidity. Concurrently, CAPE enhances MMP activity, accelerating matrix degradation and shifting the balance toward a softer, more permeable extracellular matrix. This dual mechanism may facilitate improved infiltration of effector T cells, reduce physical barriers to therapy, and dampen stromal immunosuppression. In cancer biology, ECM stiffness is increasingly recognised as a critical regulator of therapy response and immune evasion [22], and CAF heterogeneity and plasticity are central to the stromal–tumour crosstalk that shapes resistance [88]. By explicitly modelling the feedback loops between CAFs, ECM, and immune cells, our approach moves toward more physiologically grounded digital twins.

The simulation shows that CAFs and *M2* macrophages create temporary matrix stiffening (peak ∼ 30–40 h) before MMPs cause the matrix to soften. The stiffened phase shortens when cells receive CAPE treatment at higher concentrations especially when using 100 μM. The time-dependent increase in MMPs from tumour cells and CAFs becomes more rapid when cells receive CAPE treatment. The relative fold-change analysis shows that CAPE treatment reduces ECM stiffness while increasing MMP levels, which leads to a TME transition from rigid tumour-supportive to more permissive for immune cells and drug delivery. These insights resonate with emerging evidence that ECM stiffness is not merely a passive physical barrier but an active regulator of cell behaviour. Increased stiffness has been linked to enhanced tumour cell proliferation, invasion, and immune exclusion via mechanotransduction pathways (integrin–FAK, YAP/TAZ) [22,89,90].

Our model functions as a predictive framework that helps scientists design experiments to test their hypotheses about stromal remodelling and immune modulation as potential cancer treatment approaches. However, several caveats and challenges remain. Parameter calibration is only approximate, relying on assumed effect sizes and literature-derived estimates, which may limit quantitative accuracy; spatial heterogeneity and gradient effects are not captured in a well-mixed ODE framework. CAF populations are highly heterogeneous in vivo, with distinct subtypes (myofibroblastic, inflammatory, antigen-presenting), and our single-CAF model cannot resolve this variability [91]. Moreover, we lack direct empirical measurements of microenvironment metrics (e.g., ECM stiffness, MMP levels) in our PA-1/CAPE in vitro system to validate the model predictions.

The simulation results from Equations (10)–(12) provide mechanistic support for CAPE’s ability to disrupt the hypoxia–angiogenesis axis in ovarian cancer microenvironments. In our model, tumour expansion drives increasing HIF-1α levels, which in turn stimulates VEGF production, and leads to the formation of abnormal, leaky vasculature. CAPE dose-dependently suppressed each link in this chain: higher doses yielded lower steady-state HIF, reduced VEGF accumulation, and attenuated vessel formation. This modelled behaviour is consistent with the central role of HIF in coordinating hypoxic responses in tumours. HIF-1α is widely recognised as a key regulator of angiogenesis, metabolic reprogramming, and immune evasion in cancer [92]. Research studies have established that HIF activates VEGF production in different cancer types [93]. The clinical research shows that elevated HIF and VEGF levels in ovarian cancer lead to more aggressive disease and worse patient outcomes [94]. The simulation results match the expected biological responses. More interestingly, CAPE’s suppression of vessel formation in our model suggests it may not only reduce angiogenic signalling but also hamper vascular remodelling. Anti-angiogenic therapies targeting VEGF are already in clinical use (e.g., bevacizumab in ovarian cancer) and underscore the therapeutic importance of modulating vasculature [95]. Our model implies that CAPE could synergise with or act as a milder alternative to canonical VEGF inhibition by upstream modulation of HIF and downstream suppression of vessel formation.

Although our digital twin model predicts a strong anti-angiogenic effect of CAPE in ovarian cancer, available experimental data directly linking CAPE with modulation of the HIF-1α/VEGF-A axis in ovarian cancer remain lacking. To date, experimental evidence for CAPE’s anti-angiogenic activity stems largely from non-ovarian cancer systems: CAPE has been shown to inhibit VEGF-induced VEGFR-2 tyrosine-phosphorylation in endothelial cells, thereby suppressing VEGF/VEGFR-2-dependent angiogenesis [96]. Similarly, in hypoxia-challenged human retinal pigment epithelial cells, CAPE reduced VEGF secretion via downregulation of ROS, PI3K and HIF-1α signalling [61,96]. Conversely, at least one report even indicated that CAPE might activate HIF-1α (at low, non-cytotoxic doses) under certain conditions, possibly complicating a general anti-angiogenic interpretation [58,97]. Crucially, we found no studies assessing the effect of CAPE on HIF-1α or VEGF expression or signalling in ovarian cancer cell lines or tumour models. Meanwhile, it is well documented that in ovarian cancer, upregulation of HIF-1α and VEGF-A correlates with tumour angiogenesis, aggressiveness and poor prognosis [98,99]. Thus, while CAPE’s anti-angiogenic potential is supported by mechanistic data in other systems, extrapolation to ovarian cancer remains speculative, and direct experimental evidence is absent. Accordingly, our digital twin prediction should be viewed as a hypothesis—biologically plausible, but not yet validated in the relevant disease context. We therefore propose that future experimental work is needed (e.g., treatment of ovarian cancer cell lines and endothelial co-cultures under hypoxia) to test whether CAPE indeed suppresses HIF-1α stabilisation and VEGF-A production in ovarian cancer. Therefore, this illustrates the strength of computational models: they generate new hypotheses that are worth testing experimentally, which will guide our future work.

**Figure 9:**
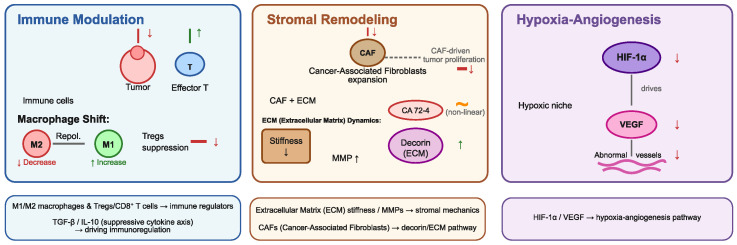
Conceptual representation of the tumour microenvironment (TME) under exposure to caffeic acid phenethyl ester (CAPE).

We present our simulation results in the schematic in Figure 9.

However, there are caveats. The model treats vasculature as a homogeneous “abnormal vessel index” without spatial heterogeneity, perfusion gradients, or vessel maturity states. In vivo, for example, vessel normalisation dynamics (i.e., pruning, pericyte coverage) and feedback with oxygen delivery complicate simple linear coupling. Also, the parameterisation of CAPE’s impact on HIF induction and degradation is hypothetical; therefore, the empirical calibration is necessary to confirm magnitudes of effect. Moreover, the effect of CAPE on endothelial cells and stromal cells is not explicitly modelled here; and real tumour vasculature responds to haemodynamic forces, shear stress, and tumour–stromal cross-signalling that are beyond the current ODE abstraction; all of that above introduce the limitation of performed simulation.

The simulation results confirm that CAPE functions as an anti-angiogenic agent through its ability to reduce the activity of the hypoxia–VEGF pathway. The combination of our previous ECM stiffness and immune cell modules with this simulation indicates CAPE creates a reprogrammed ovarian tumour environment that hinders tumour survival while making it more susceptible to immune cells and therapeutic agents. The future research should include spatial vascular models that simulate diffusion and perfusion processes to validate experimental results by measuring HIF and VEGF levels and vessel density in 3D tumour models treated with CAPE.

The ODE-based digital twin model was developed using biologically plausible parameters derived from published literature ranges. However, these parameters were not empirically calibrated to experimental time-series data. As a result, while the model captures general qualitative dynamics of the tumour microenvironment, quantitative accuracy remains limited. Future work will focus on refining parameter estimation through fitting to experimentally derived kinetic datasets and validating model outputs against in vitro and in vivo measurements. Specifically, the study measured CA72-4 and decorin only through experimental methods. We chose these two markers because they represent the main pathways for mucin production and extracellular matrix (ECM) structure changes. The current study validates its main computational results through CA.72-4 and decorin measurements, but future research could add, e.g., TGF-β, VEGF and IL-10 to achieve complete experimental verification of the digital twin model.

## 5. Conclusions

The studies conducted confirm that CAPE inhibits the proliferation and survival of ovarian teratocarcinoma cells in a dose-dependent manner. The elimination of cancer cells promotes increased presentation of immunogenic antigens to antigen-presenting cells (APCs) and directly to T lymphocytes, resulting in a strengthened immune response. Consequently, CAPE activates cytotoxic T lymphocytes and *M1* macrophages, which play a key role in controlling tumour progression.

Furthermore, the data obtained indicate that CAPE can modulate the biochemistry of the ovarian cancer microenvironment by stimulating the synthesis of cytokines and chemokines that activate immunocompetent cells and by reducing the concentrations of metabolites that promote intense cancer cell proliferation, tissue remodelling and neovascularisation.

It should be emphasised that the system of interactions between cells in the tumour niche is highly complex, dynamic and varies from patient to patient, which justifies the need for further research to unequivocally confirm the mechanisms presented.

In this context, the use of artificial intelligence tools and digital twin models seems particularly promising, as it enables broad interpretation of large molecular, biochemical and clinical datasets, as well as prediction of treatment response and risk of disease recurrence. In addition, virtual modelling significantly reduces the costs and time required to conduct classic experimental research, which gives hope for rapid progress in cancer diagnosis and treatment.

Our digital twin outputs should be interpreted as testable hypotheses: mechanistically plausible but not yet confirmed within the appropriate disease model. Accordingly, additional experimental studies will be required to verify these predictions.

## Figures and Tables

**Table 1:** Parameters used in the ODE-based CAPE digital twin (Equation (3)).

Parameter	Meaning	Unit	Value and Source
*r*	Tumour intrinsic growth rate	h^−1^	0.05 [27]
*K*	Tumour carrying capacity	cells	$1\times 10^{6}$ [28]
α	Effector T-cell tumour-killing coefficient	(cells·h)^−1^	$1\times 10^{-7}$ [27]
*s*	Effector T-cell stimulation rate	cells·h^−1^	100 [29]
$K_{E}$	Half-saturation constant for T-cell activation	cells	$1\times 10^{5}$ [30]
$d_{E}$	Effector T-cell natural decay rate	h^−1^	0.05 [27]
δ	CAPE-induced tumour suppression coefficient	h^−1^	{0,0.02,0.05,0.08,0.1} (dose–response mapping) This study, calibrated [8]

Values correspond to MATLAB implementation used for CAPE–TME simulations.

**Table 2:** Parameters used in the macrophage (M1/M2) and Treg dynamics, Equations (4)–(6).

Parameter	Meaning	Unit	Value and Source
$r_{M1}$	Baseline *M1* macrophage growth rate	h^−1^	0.02 [31]
$r_{M2}$	Baseline *M2* macrophage growth rate	h^−1^	0.02 [32]
*K*	Carrying capacity for macrophages (shared niche constraint)	cells	$1\times 10^{6}$ [28]
α	Suppressive effect of Tregs on *M1* population	(cells·h)^−1^	0.001 [33]
β	Promotion of *M2* macrophages by Tregs	(cells·h)^−1^	0.001 [34]
γ	Lactate-driven stabilisation/expansion of *M2* macrophages	h^−1^	0.002 [35]
$s_{R}$	Baseline source rate of Tregs	cells·h^−1^	0.01 [36]
$\alpha_{R}$	Treg expansion induced by suppressive cytokines (TGF-β/IL-10)	h^−1^	−(implicit in code via *M2*→ Treg term) [37]
$\beta_{R}$	Lactate-driven stabilisation/metabolic support of Tregs	h^−1^	−(implicit in code via *M2*→ Treg term) [38]
$\delta_{M1}$	Natural decay rate of *M1* macrophages	h^−1^	0.01 (derived from model structure) [39]
$\delta_{M2}$	Natural decay rate of *M2* macrophages	h^−1^	0.01 (derived from model structure) [40]
$\delta_{R}$	Natural decay rate of regulatory T cells	h^−1^	0.005 (model value) [41]

Values correspond to MATLAB implementation used for CAPE–TME simulations.

**Table 3:** Parameters used in the CAF–tumour–cytokine module (Equation (7) and coupled feedback terms).

Parameter	Meaning	Unit	Value and Source
$s_{F}$	Baseline source rate of cancer-associated fibroblasts (CAFs)	cells·h^−1^	5 [42]
$\alpha_{F}$	Tumour-driven CAF induction rate (cancer cells → CAF activation)	h^−1^ (per tumour cell, scaled)	$1\times 10^{-4}$ [43]
$\gamma_{F}$	Cytokine/TGF-β–driven CAF induction rate	h^−1^ (per cytokine unit, scaled)	$1\times 10^{-3}$ [44]
$d_{F}$	Natural decay/turnover rate of CAFs	h^−1^	0.01 [45]
$k_{F}^{\mathrm{CAPE}}$	Strength of CAPE-mediated suppression of CAFs (applied via Hill function f(D))	h^−1^	Calibrated in this study (initial guess $1\times 10^{-3}$; final value obtained by fitting CAF (72 h, 100 µM) ≈ 0.5× control)
IC_50_	Half-maximal inhibitory concentration for CAPE in CAF suppression Hill function f(D)	μM	50 Chosen in the mid–μM range to reflect experimentally observed CAPE doses with ∼50% effect on stromal readouts
$n_{H}$	Hill coefficient for CAPE response (cooperativity of suppression)	dimensionless	2 [46]
$r_{T}$	Baseline tumour proliferation rate (before CAF boost)	h^−1^	0.02 [28]
$K_{T}$	Tumour carrying capacity in this module	cells	$1\times 10^{6}$ Standard logistic carrying capacity scale for in vitro tumour cell populations in ODE models [28]
$\beta_{F}$	Strength of CAF-induced boost to tumour proliferation ($r_{T,\mathrm{eff}}=r_{T}\left(1+\beta_{F}F/\left(K_{F}+F\right)\right)$)	dimensionless	1.0 [47]
$K_{F}$	Half-saturation constant for CAF effect on tumour growth	cells	$1\times 10^{4}$ Sets the CAF scale at which tumour proliferation is approximately half-maximally boosted (this study; guided by CAF density ranges in [42,43])
$\alpha_{C}$	Tumour-driven cytokine production rate (e.g., IL-6/TGF-β–like signals)	cytokine units·h^−1^ (scaled)	$1\times 10^{-4}$ [48]
$d_{C}$	Cytokine clearance/decay rate	h^−1^	0.01 [48]

Values correspond to MATLAB implementation used for CAPE–TME simulations.

**Table 4:** Parameters for extracellular matrix (ECM) stiffness (Equation (8)) and matrix metalloproteinase (MMP) dynamics (Equation (9)) with CAPE modulation.

Parameter	Meaning	Unit	Value and Source
$\rho_{S}$	CAF-driven ECM phenomenological rate capturing the ability of CAFs to deposit and cross-link ECM, increasing stiffness. Values chosen to give slow but progressive stiffening over 72–200 h, consistent with CAF–ECM coupling.	a.u.·h^−1^	$1\times 10^{-3}$ [49]
$\lambda_{S}$	*M2* macrophage-driven ECM stiffening rate (phenomenological)	a.u.·h^−1^	$1\times 10^{-3}$
$\delta_{S}$	Baseline ECM relaxation/softening rate (phenomenological); models spontaneous loss of stiffness in absence of continued CAF/*M2* input; relatively slow decay	h^−1^	$5\times 10^{-3}$
$\delta_{S}^{\prime }$	MMP-mediated ECM degradation coefficient (stiffness loss per unit MMP)	(a.u.·h)^−1^	$1\times 10^{-4}$ [49]
$\alpha_{\mathrm{MMP},T}$	Tumour-driven MMP production rate	a.u.·h^−1^	$1\times 10^{-4}$ [50]
$\alpha_{\mathrm{MMP},F}$	CAF-driven MMP production rate	a.u.·h^−1^	$1\times 10^{-4}$ [51]
$\mu_{\mathrm{MMP}}$	Baseline MMP decay/inactivation rate	h^−1^	$1\times 10^{-2}$ [52]
$k_{\mathrm{MMP}}^{\mathrm{prod}}$	CAPE effect on CAF-driven MMP production (weakening factor). Value calibrated phenomenologically to reflect experimental tendency of CAPE and related polyphenols to reduce MMP expression.	dimensionless	0.5
$k_{\mathrm{MMP}}^{\deg}$	CAPE effect on MMP degradation (strengthening factor). Chosen to complement the production effect and mimic reported MMP-suppressive actions of phytochemicals.	dimensionless	0.5 [53]
IC_50_	Half-maximal CAPE concentration in Hill function f(D), aligned with typical ranges for CAPE’s antiproliferative and anti-invasive effects in vitro.	μM	50
$n_{H}$	Hill coefficient for CAPE response (cooperativity). Moderately sigmoidal CAPE response; widely used for cooperative pharmacodynamic effects and matches earlier modules in this study.	dimensionless	2

Values correspond to MATLAB implementation used for CAPE–TME simulations.

**Table 5:** Parameters for hypoxia–HIF–tumour coupling (Equation (10)) with CAPE modulation, including biological meaning, units, MATLAB-implemented values and literature justification.

Parameter	Meaning	Unit	Value and Source
$\alpha_{H}$	Basal HIF-1α induction rate (hypoxia signalling strength)	h^−1^	0.05 [54]
$K_{O_{2}}$	Half-saturation constant for oxygen-dependent HIF suppression	mmHg (scaled)	20 [55]
$K_{TH}$	Saturation constant for tumour-driven HIF activation	cells	$1\times 10^{5}$ [56]
$\delta_{H}$	HIF-1α degradation rate	h^−1^	0.01 [57]
$O_{2,\mathrm{base}}$	Baseline tissue oxygen level	mmHg (scaled)	10 [55]
IC_50_	Half-maximal CAPE concentration in Hill function f(D), aligned with typical ranges for CAPE’s antiproliferative and anti-invasive effects in vitro.	μM	50
$n_{H}$	Hill coefficient for CAPE response (cooperativity). Moderately sigmoidal CAPE response; widely used for cooperative pharmacodynamic effects and matches earlier modules in this study.	dimensionless	2
$k_{O_{2}}$	CAPE-induced relative increase in effective oxygenation (phenomenologically)	dimensionless	0.60
$k_{\alpha }$	CAPE-induced reduction in HIF induction	dimensionless	0.40 [58]
$k_{\delta }$	CAPE-induced increase in HIF degradation	dimensionless	0.50 [58]
$k_{rT}$	CAPE-induced reduction in tumour proliferation rate	dimensionless	0.35 calibrated to [59]
$k_{\mathrm{kill}}$	CAPE-dependent direct tumour-killing coefficient (phenomenologically)	h^−1^	0.015

Values correspond to MATLAB implementation used for CAPE–TME simulations.

**Table 6:** Parameters for VEGF (Equation (11)) and abnormal vessel density (Equation (12)) in the hypoxia–angiogenesis module.

Parameter	Meaning	Unit	Value and Source
$\alpha_{V}$	HIF-driven VEGF production rate	h^−1^	0.10 [60]
$\delta_{V}$	VEGF degradation/clearance rate	h^−1^	0.02 [61]
$\alpha_{A}$	VEGF-induced abnormal vessel growth rate	h^−1^	0.05 [62]
$K_{VA}$	Half-saturation constant for VEGF-driven vessel growth	a.u. (VEGF)	50 [60]
$A_{\max}$	Carrying capacity for abnormal vessel density	a.u.	100 [63]
$\delta_{A}$	Abnormal vessel regression/pruning rate	h^−1^	0.01 [60]
IC_50_	Half-maximal CAPE concentration in Hill function f(D), aligned with typical ranges for CAPE’s antiproliferative and anti-invasive effects in vitro.	μM	50
$n_{H}$	Hill coefficient for CAPE response (cooperativity). Moderately sigmoidal CAPE response; widely used for cooperative pharmacodynamic effects and matches earlier modules in this study.	dimensionless	2

Values correspond to MATLAB implementation used for CAPE–TME simulations.

## Data Availability

The code used for this paper is available at Zenodo with the DOI: https://doi.org/10.5281/zenodo.17422069.

## Appendix A. Statistical Analysis

**Table A1:** Welch ANOVA results for CAPE-induced modulation of CA72-4 and decorin levels at 24 h and 48 h in PA-1 cells. Welch’s heteroscedastic ANOVA was used due to unequal variances and unbalanced variance structure (*** p<0.001).

Biomarker	Time Point	Welch *F*	*p*-Value
CA72-4	24 h	53.15	3.71 $\times 10^{-10}$ ***
CA72-4	48 h	459.46	3.51 $\times 10^{-18}$ ***
Decorin	24 h	39.08	0.000301 ***
Decorin	48 h	776.07	1.17 $\times 10^{-7}$ ***

**Table A2:** Dunn’s post hoc comparison vs. control for CA72-4 and decorin following CAPE treatment. Holm-adjusted *p*-values are shown. Statistical differences vs. control were determined using the Kruskal–Wallis test with Dunn’s post hoc test (** p<0.01, *** p<0.001), n.s.—not significant.

Biomarker	Dose (μM)	*p*-Value	Significance
CA72-4 (24 h)	Control	—	—
	10	0.00544	**
	25	0.00453	**
	50	0.00363	**
	100	0.00272	**
	200	0.00181	***
CA72-4 (48 h)	Control	—	—
	10	0.00544	**
	25	0.00453	**
	50	0.00363	**
	100	0.00272	**
	200	0.00181	**
Decorin (24 h)	Control	—	—
	10	0.10	n.s.
	25	0.10	n.s.
	50	0.00455	**
	100	0.00378	**
	200	0.00132	**
Decorin (48 h)	Control	—	—
	10	0.10	n.s.
	25	0.10	n.s.
	50	0.00433	**
	100	0.00204	**
	200	0.00303	**
